# Effects of bee venom and dopamine-loaded nanoparticles on reserpine-induced Parkinson’s disease rat model

**DOI:** 10.1038/s41598-021-00764-y

**Published:** 2021-10-27

**Authors:** Omar A. Ahmed-Farid, Mohamed Taha, Rofanda M. Bakeer, Omyma K. Radwan, Hassan A. M. Hendawy, Ayman S. Soliman, Einas Yousef

**Affiliations:** 1 Physiology Department, National Organization for Drug Control and Research (NODCAR), Giza, Egypt; 2 Biochemistry Department, Faculty of Pharmacy, Cairo University, Giza, Egypt; 3 Pathology Department, Faculty of Medicine, Helwan University, Helwan, Egypt; 4 Analytical and Inorganic Chemistry Department, NODCAR, Giza, Egypt; 5 Medical Physiology Department, Faculty of Medicine, Beni-Suef University, Beni-Suef, Egypt; 6 Basic Medical Sciences Department, College of Medicine, Dar Al Uloom University, Riyadh, Kingdom of Saudi Arabia; 7 Histology and Cell Biology Department, Faculty of Medicine, Menoufia University, Shibin El Kom, Egypt

**Keywords:** Biochemistry, Physiology, Medical research, Nanoscience and technology

## Abstract

Parkinson’s disease (PD) is a progressive chronic neurodegenerative condition characterized by the loss of dopaminergic neurons within the substantia nigra. Current PD therapeutic strategies are mainly symptomatic and can lead to motor complications overtime. As a result, alternative medicine may provide an effective adjuvant treatment for PD as an addition to or as a replacement of the conventional therapies. The aim of this work was to evaluate the effects of Bee Venom (BV) and dopamine (DA)-loaded nanoparticles in a reserpine-induced animal model of PD. After inducing PD with reserpine injection, different groups of male rats were treated with L-Dopa, BV, DA-nanoparticles. Our findings showed that BV and DA-nanoparticles administration restored monoamines, balanced glutamate/GABA levels, halted DNA fragmentation, decreased pro-inflammatory mediators (IL-1β and TNF-α), and elevated anti-inflammatory mediators (PON1) and neurotropic factor (BDNF) levels in comparison with conventional therapy of PD. Furthermore, in a reserpine-induced PD rat model, the ameliorative effects of BV were significantly superior to that of DA-nanoparticles. These findings imply that BV and DA-nanoparticles could be useful as adjuvant treatments for PD.

## Introduction

Parkinson's disease (PD) is a progressive neurodegenerative disorder that was first described as shaking palsy by James Parkinson. It is characterized by pathological brain alterations which include degeneration of dopamine secreting neurons in substantia nigra and aberrant aggregation of alpha-synuclein protein in the cytoplasm of neurons forming Lewy bodies^1^. Clinically, PD is characterized by many motor symptoms, such as slow movement, stiffness, resting tremors, imbalances, as well as some non-motor manifestations such as sleep disorders, pain, fatigue, and gastrointestinal symptoms^2,3^. The specific mechanisms driving the profound and irreversible dopaminergic neuron degeneration are still not yet understood. However, multiple cellular processes might be possibly involved in this disease such as neuroinflammation, oxidative stress and mitochondrial dysfunction^4–6^.

Currently, available therapies of PD are mainly focus on alleviating motor symptoms by compensating dopamine deficiency. Although dopamine does not cross the blood–brain barrier, its immediate precursor, levodopa, is actively transported into the central nervous system and converted to dopamine in the brain^7^. Levodopa acts by restoring dopaminergic neurotransmission in the corpus striatum by enhancing dopamine synthesis in the surviving neurons of the substantia nigra^8^. Large doses of levodopa are required as much of the drug is decarboxylated to dopamine in the periphery, resulting in adverse effects such as nausea, vomiting, cardiac arrhythmias, and hypotension^9^. Unfortunately, as the disease progress, fewer residual nigrostriatal neurons are capable of taking up and converting exogenously administered levodopa to dopamine for subsequent storage and release^10^. As a result, as the PD progresses, the majority of patients experience shorter duration of response to individual doses of levodopa and the drug effects “wear off” with subsequent motor control fluctuations such as involuntary movements of the head, trunk or limbs^11^.

Bee Venom (BV) is a colourless, acidic biological material, produced by the bees’ poison glands that has strong odour and bitter taste^12^. For thousands of years, bee venom therapy has been used in alternative medicine^13^. It has been used in the treatment of some diseases such as rheumatoid, gout, neuropathy, and inflammatory disorders. There are different methods of BV administration such as acupuncture and injections which are available in pharmacies or directly via bee stings^14^. BV contains a variety of active ingredients including proteins (phospholipase A2, phospholipase B, hyaluronidase, phosphatase α–glucosidase), peptides (melittin, apamine, mast cell degranulating peptide, adolapine, procamine A, B-protease inhibitor tertiapine, biogenic amines (histamine, dopamine, noradrenalin), amino acids (aminobutyric acid, α-amino acids), sugars (glucose, fructose), and minerals (P, Ca, Mg)^15–17^.

With the advance of medical field, new nanotechnology-based approaches have been used in drug delivery systems that can traverse the blood brain barrier^18^. Molecules and drug of interest can be coupled to these delivery systems, which can then be actively delivered to and target certain damaged tissues or organs. The primary aim of using the nanosized drug delivery system in PD is to increase the medication availability in the brain without increasing the administered dose, hence reducing drug adverse effects^19^. Cancer treatment with nanoparticles or gold shields, as well as a variety of other diseases, are some of the most important uses in the field. Nanotechnology also opens up new opportunities in implantable medication delivery systems, which are frequently favoured over injectable pharmaceuticals^20^. The goal of tis study is to assess and compare the possible effects of L-Dopa, BV, and novel self-nanoemulsifying dopamine delivery system on reserpine-induced PD rat model.

## Materials and methods

### Animals and experimental design

In the current study, we handled the animals consistently in accordance with the ARRIVE guidelines. On October 29, 2019, the institutional Ethics Committee at NODCAR and Faculty of Pharmacy, Cairo University, approved all animal procedures. All experiments were carried out in accordance with the authorized experimental protocols and the Guide for Care and Use of Laboratory animals of The National Organization for Drug Control and Research (NODCAR). For all conducted experiments, a total of 30 male rats, three-month-old were used. All rats were housed in standardized cages in groups of 2–3 per cage, in a room with controlled temperature (25 ± 1 °C), humidity and luminosity (12:12 light/dark cycle, lights on at 6:30 a.m.), with food and tap water provided ad libitum. We randomly divided the experimental rats into five groups with six animals per group as following:

- Group 1 (control) received intraperitoneal (i.p) injection of 0.5 ml saline.
- Group 2 treated with reserpine (1 mg /kg, i.p.) on day 1, 3, and 5 (alternate day) for a period of 5 days to induce PD^21,22^. Reserpine is a potent vesicular monoamine transporter 2 (VMAT2) inhibitor which used in treatment of hypertension and is known to induce depletion of central catecholamines storage. This rat model of PD is reported to exhibit muscular rigidity, tremors, and akinesia^23^.
- Group 3 treated with reserpine (1 mg /kg, i.p., on day 1, 3, and 5 (alternate day) for a period of 5 days) concurrently with L-Dopa (22.5 mg/kg oral (P.O.), daily) for 30 days^24^.
- Group 4 treated with reserpine (1 mg /kg, i.p., on day 1, 3, and 5 (alternate day) for a period of 5 days) concurrently with BV (10 μl/kg, i.p, every other day) for 30 days^25^.
- Group 5 treated with reserpine (1 mg /kg, i.p., on day 1, 3, and 5 (alternate day) for a period of 5 days) concurrently with Dopamine (DA)-nanoparticles (22.5 mg/kg P.O., daily) for 30 days.

After 24 h of last dosing of different therapies, all animals were deeply anesthetized with 2 mg pentobarbital then transcardially perfused with 200 ml of heparinized saline, followed by 250 ml of 4% paraformaldehyde (PFA) in PBS. The brain was then surgically removed for further processing.

### Materials

All chemicals, solvents and reagents of high-performance liquid chromatography (HPLC) were purchased from Sigma-Aldrich (St. Louis, MO, USA). Bee venom was purchased from local markets and authenticated at the Faculty of Agriculture, Benha University. Reserpine was purchased from Novartis. All other chemicals and solvents used were of analytical grade and were utilized as received from the manufacturer without further purification.

### Preparation of Dopamine (DA)-loaded nanoparticles

To prepare self-nanoemulsifying drug delivery systems, isotropic mixtures of DA and soy lecithin were dissolved in peanut oil with the addition of chloroform to achieve an oily phase. The chloroform was removed using oven under vacuum. To obtain nano-sized emulsion, Tween 80 was added to the oily phase and ultrasonicated using an ultrasonicator. The nano-emulsion obtained was filtered using a 0.22 µm membrane.

### Morphological characterization and size distribution of nanoparticles

The prepared optimized DA-nanoparticles formulations were characterized for particle size, Polydispersity Index (PDI) and Zeta potential using Zetasizer. For assessing the relevant parameters of the formulations, the average particle count rate was kept between 30 and 80 kcps. Assessment of the shape and surface morphology were conducted using the Transmission Electron Microscopy (TEM). One drop of the nano-emulsion was placed on copper grids and stained with 2% (w/v) phosphotungstic acid for 5 min at room temperature. Finally, images were produced by employing a TEM instrument (Tecnai-12, Philips) to observe the grids bearing nanoemulsion. Mean diameter and size distribution of nanoparticles were determined using a Zetatrac Ultra at 25 °C. Volume-distribution curves were used to represent the size distribution.

### Sample collection and preparation

For neurochemical analysis, brains of the experimental rats were quickly removed from the cranium and immediately frozen on dry ice and stored at − 80 °C until use. Isolation of selected brain structure (the hippocampus and striatum) was done using brain matrix according to The Rat Brain Atlas^26^. The collected hippocampus and striatum were weighed and homogenized in 75% aqueous HPLC grade methanol (10% w/v). The homogenate was spun for 15 min at 4000 r.p.m., the supernatant was isolated, filtered and used for the HPLC as described in the next section.

### High performance liquid chromatography (HPLC)

Using the HPLC technique, neurochemical analysis of brain tissues was performed to evaluate brain monoamines (Norepinephrine, Dopamine, Serotonin (5-HT)) and free amino acids, as described by Heinrikson and Meredith (1984) and Pagel et al. (2000)^27,28^. The activity of acetylcholinesterase (AChE) in brain tissue homogenate was assessed by the HPLC, as described by Gorun et al. (1978)^29,30^.

### Brain paraoxonase-1 (PON1) activity

The activity of PON1 was measured using the method of Gatica et al. (2006)^31^. This assay involves measuring the hydrolysis of phenylacetate (substrate) by PON1/arylesterase activity releasing phenol. The phenol produced after the addition of a 40-fold diluted homogenate sample was spectrophotometrically measured at 217 nm. Blanks were included to correct the spontaneous hydrolysis of phenylacetate. The activity of PON1 was expressed in Kunit/g wet tissue. The enzyme quantity that disintegrates 1 nmol phenylacetate per minute was specified as one unit.

### Derivatization procedure

Standards and samples were derivatized using the following solutions. Drying solution was prepared as a 2:2:1 mixture (by volume) of methanol: 1 M sodium acetate trihydrate: triethylamine (TEA). The drying solution was applied to the dry samples, shook well and vacuum dried until completely dry. The derivatizing agent consisted of 7:1:1:1 (by volume) mixture of methanol: TEA: double distilled deionized water: PITC. The derivatizing solution was added to the re-dried sample, shook well and set aside for 20 min at room temperature before being dried under vacuum (70 millitorr). The dry sample was then diluted with a sample diluent made up of 0.71 g disodium-hydrogen phosphate that had been adjusted to a pH of 7.4 with 10% phosphoric acid. Acetonitrile was then mixed, as 5% by volume with the resulting solution. For analysis, a 20 μL aliquot of the derivatized standards and samples mixture was injected into the HPLC system.

### Enzyme-linked immunosorbent assay (ELISA) of brain antioxidants and inflammatory mediators

Serum Interleukin 1 β (IL-1β) levels (pg/ml), tissue tumour necrosis factor alpha (TNF-α) and brain-derived neurotrophic factor (BDNF) (pg/g) were determined by ELISA kit (RayBio ® Rat IL-1β USA), (Rat TNF-α ELISA Kit Koma Biotech Inc, Korea), and (Glory Science Co., Ltd, Del Rio, TX, USA) respectively according to Sakamoto et al. (1994)^32^. The manufacturer's instructions (Invitrogen) were followed and the plate was read using a micro plate reader (Biotech ELx800) set at 450 nm. The sample concentration was calculated against a standard curve and is reported in nanograms (ng).

### DNA Comet assay

Comet assay of DNA was estimated according to the classic alkaline single-cell electrophoresis protocol to detect DNA damage^33^. The cells were embedded in agarose on a slide and subjected to lysis and electrophoresis under controlled condition. During electrophoresis, the fragmented negatively charged DNA migrated away from the nucleus to the anode. The extend of DNA damage was detected by assessing the amount of migrated DNA. Samples were stained with ethidium bromide (“Sigma”, Germany), examined by fluorescence microscopy and analysed by Comet Score 1.5 software to detect DNA. Two slides were prepared for each condition. Cells with damaged DNA had the appearance of comet with a bright head and tail. Unaffected cells with undamaged tightly coiled DNA appear to have intact nuclei with no tail. The comet parameters considered in this study were % of tailed DNA, tail length, tail intensity (% of DNA in the tail), and the tail moment. The percent of DNA in comet tails was considered as a genotoxic effect marker.

### Statistical analysis

All data sets were tested for normality distribution with the Shapiro–Wilk test prior to any statistical analysis. The data of our samples were expressed as mean ± SE. The differences between two groups were analysed by one-way ANOVA using the general linear model (GLM) produced by Statistical Analysis Systems Institute (SAS, 1989). Significant differences among means were evaluated using Duncan’s Multiple Range Test of SAS (1989). A *p*‐value < 0.05 was considered statistically significant.

## Results

### Morphological characterization and size distribution

**Figure 1:**
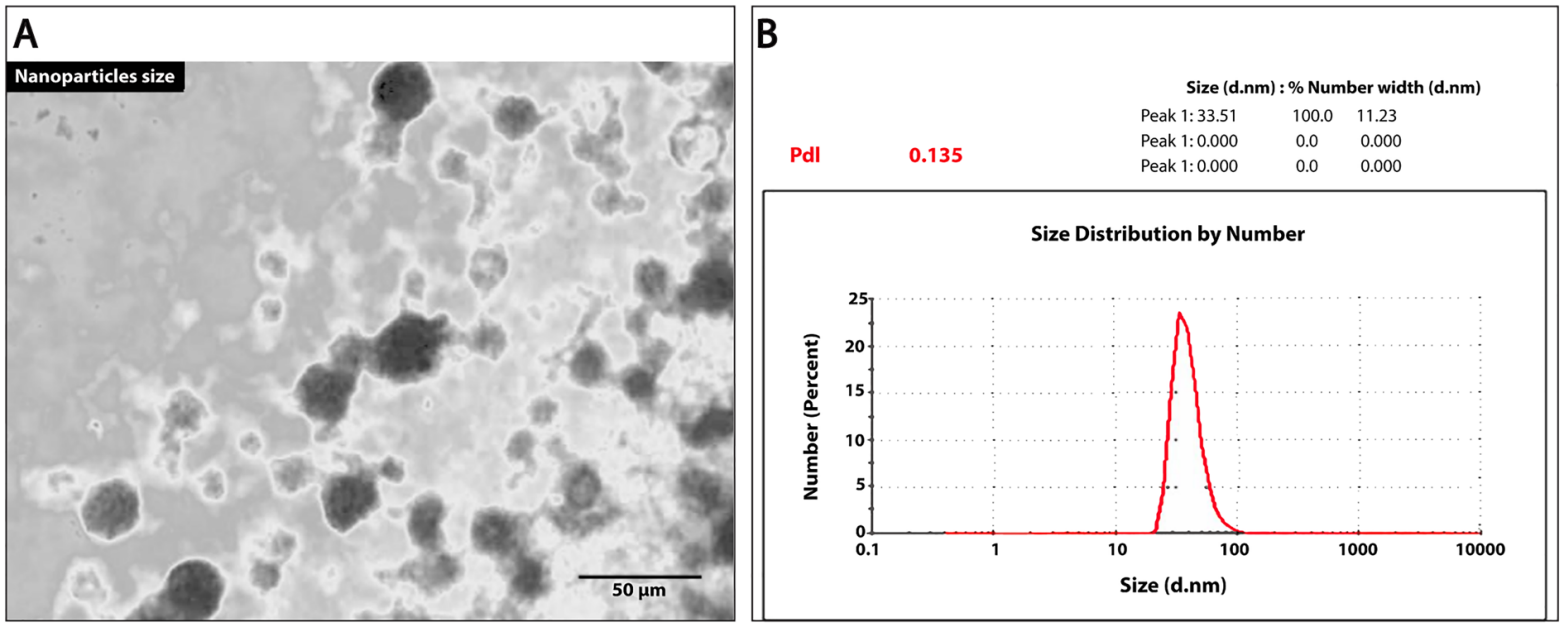
(**A**) Transmission electron microscopy images of self-nanoemulsifying dopamine delivery system nanoparticles synthesized on this study. Dopamine nanoform of 32.6: 48.5 nm, particles perceived to be spherical in shape. (**B**) Size distribution and zeta potential of DA-nanoparticles were determined using light scattering (DLS).

Determination of the zeta potential of DA-nanoparticles is extremely valuable as it provides an idea about the prepared suspension stability. It was observed that the prepared DA- nanoparticles remained stable for more than several weeks, with no signs of aggregation or precipitation. In our case, the values of zeta potential for the prepared nanoparticles with respect to time are specified in (Table 1). In different times, the zeta potential ranged from 10.4 to 12.0 nm which is adequate for crossing the blood brain barrier. The prepared DA-nanoparticles preserved their characteristics when stored at room temperature. When the temperature was increased from 4 to 50 °C over a period of 90 days, the hydrodynamic size of the nanoparticles remained stable. However, when the temperature was raised after 90 days, the nanoparticles agglomerated indicating the possibility of storing this formula at room temperature (Fig. 1A,B).

**Table 1:** Variations in DA-nanoparticles characteristics with respect to time.

Property time	Day 1	After 15 days	30 days	60 days
Size (nm)	10.4 ± 2.9	12.0 ± 2.2	11.2 ± 2.9	12.0 ± 2.9
ZP	23.0 ± 2.8	23.0 ± 2.8	23.0 ± 2.8	23.0 ± 2.8

Data are expressed as Mean ± S.E.

### Reserpine-induced neurotransmitters chemical changes in the brain tissues of rat model of PD

**Figure 2:**
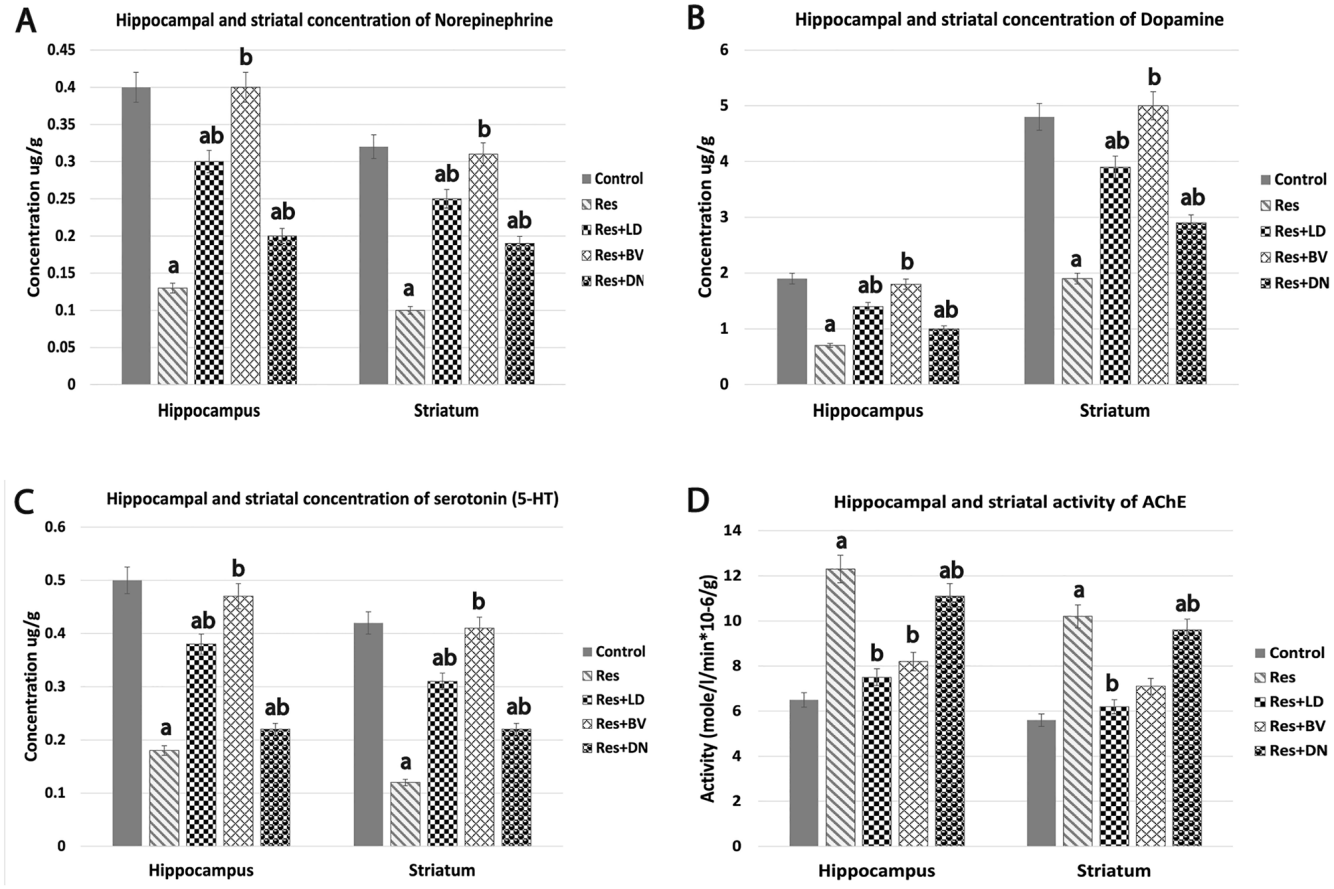
HPLC analysis of the effects of L-Dopa, BV, and DA nanoparticles on hippocampus and striatal monoamines, as well as AChE activity, in a reserpine-induced Parkinson's rat model. (**A**) Norepinephrine, (**B**) Dopamine, (**C**) Serotonin (5-HT), and (**D**) AChE. Data are expressed as Mean ± S.E. for 6-rats/group. a indicates significant difference from control group (*p* < 0.05), b indicates significant difference from the reserpine-treated group, ab indicates significant difference from both control and reserpine-treated group.

Hippocampal and striatal levels of three monoamines neurotransmitters (norepinephrine, dopamine, serotonin (5-HT)) and acetylcholinesterase (AChE), as indicator of acetylcholine level, were determined by HPLC in homogenized tissues of isolated brains after reserpine administration (1 mg/kg, i.p., every other day, for 5 days). When compared to the control group, the one-way ANOVA revealed that reserpine induced a significant reduction (*p* < 0.05) in hippocampal and striatal norepinephrine, dopamine, serotonin and acetylcholine, as determined by significant elevation of AChE levels (Fig. 2A–D).

Using the HPLC, we investigated the effect of reserpine administration on the levels of four amino acid neurotransmitters in hippocampal and striatal tissues: Gamma-Aminobutyric Acid (GABA), Arginine (Arg), Histidine (His), and Glutamate (Glu). In comparison to the control group, reserpine administration induced significant reduction in the levels of GABA, a well-known inhibitory neurotransmitter, and Arg in both hippocampal and striatal tissues. Furthermore, significant elevated levels of His and Glu, well-known excitatory neurotransmitters, were detected in rats treated with reserpine in both hippocampal and striatal tissues in comparison to control group (Table 2).

**Table 2:** Effect of L-Dopa, BV, and DA-nanoparticles on brain amino acids neurotransmitters in reserpine-treated rats.

Groups		Parameters			
		GABA	Arg	His	Glu
Hippocampus	Control	6.305 ± 0.216	3.289 ± 0.1	0.939 ± 0.029	4.054 ± 0.125
	Res	2.016 ± 0.07a	1.064 ± 0.034a	1.532 ± 0.047a	6.513 ± 0.231a
	Res + LD	4.805 ± 0.159ab	2.508 ± 0.085b	1.072 ± 0.036	4.609 ± 0.158b
	Res + BV	4.932 ± 0.159ab	3.173 ± 0.111b	1.198 ± 0.042	4.876 ± 0.163b
	Res + DN	3.171 ± 0.102a	1.673 ± 0.051a	1.423 ± 0.05	6.109 ± 0.195a
Striatum	Control	5.227 ± 0.168	2.76 ± 0.091	0.789 ± 0.025	3.479 ± 0.116
	Res	1.753 ± 0.062a	0.933 ± 0.029a	1.336 ± 0.042a	5.707 ± 0.173a
	Res + LD	4.149 ± 0.143ab	2.033 ± 0.071b	0.934 ± 0.029	3.779 ± 0.125b
	Res + BV	3.774 ± 0.127ab	2.96 ± 0.097b	1.055 ± 0.037	4.26 ± 0.138ab
	Res + DN	2.757 ± 0.09a	1.419 ± 0.048	1.151 ± 0.04	5.278 ± 0.178a

Data are expressed as Mean ± S.E. for 6-rats/group.

a letter marks significant difference from control group at the same column with one-way ANOVA at *P* < 0.05.

b letter marks significant difference from reserpine group at the same column with one-way ANOVA at *P* < 0.05.

ab indicates significant difference from both control and reserpine-treated group.

### Effects of L-Dopa, BV, and DA-nanoparticles on reserpine-induced neurotransmitters chemical changes in brain tissues of PD rat model

A. ***Effects on neurotransmitters***

Next, we assessed the effect of L-Dopa, BV, and DA-nanoparticles administration on the reserpine-induced neurotransmitter chemical alterations in hippocampal and striatal tissues using HPLC in homogenized tissues from respective experimental rats. It was demonstrated that DA-nanoparticles is an effective tool of sustained and safe delivery of dopamine across the blood brain barrier. These nanoparticles release dopamine slowly and constantly, reduce the plasma dopamine clearance, quinone adduct formation and dopamine autoxidation^34^. When compared with the reserpine-treated group, one-way ANOVA indicated significantly higher levels (*p* < 0.05) of hippocampal and striatal monoamines neurotransmitters (norepinephrine, dopamine, serotonin (5-HT)), and reduced activity of AChE in rats treated with L-Dopa, BV, and DA-nanoparticles. Indeed, our findings revealed that BV administration (10 μl/kg, i.p., every other day, for 30 days) was able to restore the three monoamines and reduced AChE activity to near-normal levels in both hippocampus and striatum, with no significant difference between BV-treated rats and control group for any of the assessed monoamine neurotransmitters (Fig. 2A–D).

To further understand the effects of L-Dopa (22.5 mg/kg, P.O.), BV (10 μl/kg b.w i.p.), and DA-nanoparticles (22.5 mg/kg P.O.) administration for 30 days on reserpine-induced chemical changes in the brain, we assessed the effects of these treatments on the levels of hippocampal and striatal GABA, Arg, Glu and His. Our results from studying the hippocampal tissue revealed significant elevation of inhibitory amino acids neurotransmitters (GABA and Arg) upon administration of both L-Dopa and BV when compared with reserpine-treated group. Of note, no significant difference was detected in rats treated with DA-nanoparticles when compared with reserpine-treated group. When comparing the excitatory amino acid neurotransmitters, we found that animals treated with L-Dopa and BV had much lower levels of Glu than rats treated with reserpine. Similar results were obtained from studying the striatal tissues (Table 2). It is worth noting that neither treatment affected His levels. Our findings show that both L-Dopa and BV therapies may be able to rescue the brain form the detrimental effects of excess glutamate in PD. Furthermore, when it comes to guarding against reserpine-induced neurotoxicity, both L-Dopa and BV outperform DA-nanoparticles.

B. ***Effects on brain inflammatory mediators***

**Figure 3:**
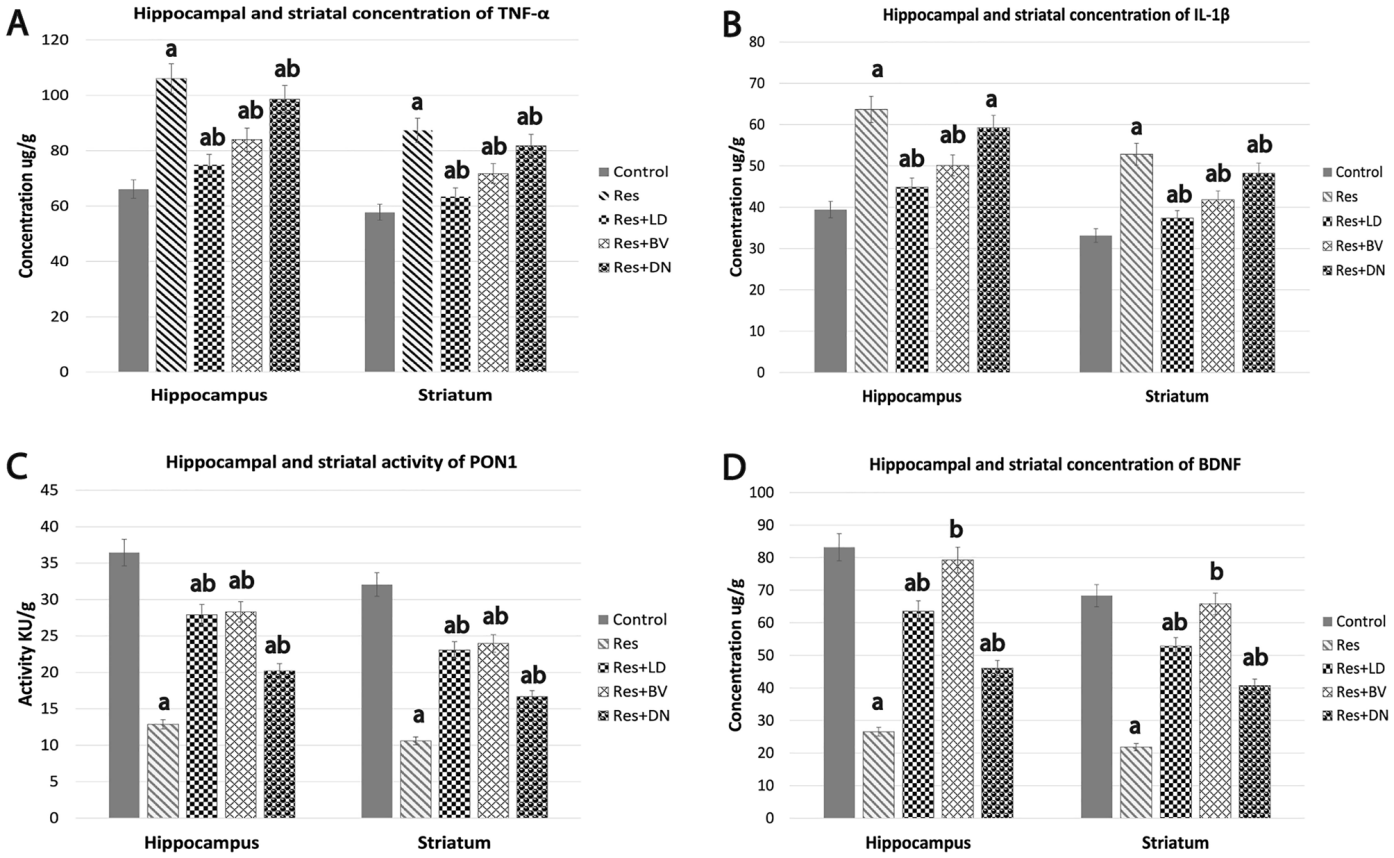
HPLC analysis of the effects of L-Dopa, BV, and DA nanoparticles on hippocampus and striatal inflammatory mediators in a reserpine-induced Parkinson's rat model. (**A**) Tumour necrosis factor alpha (TNF-α), (**B**) Interleukin 1 β (IL-1β), (**C)** Paraoxonase-1 (PON1), and (**D**) Brain-derived neurotrophic factor (BDNF). Data are expressed as Mean ± S.E. for 6-rats/group. a indicates significant difference from control group (*p* < 0.05), b indicates significant difference from the reserpine-treated group (*p* < 0.05), ab indicates significant difference from both control and reserpine-treated group.

We next investigated the effect of the three treatments on the brain pro- and anti-inflammatory mediators in reserpine-induced PD in both hippocampus and striatum using HPLC in homogenized tissues from respective experimental rats. In comparison to the control group, reserpine treated rats had significantly high levels of proinflammatory mediators (TNF-α and IL-1β), and significantly lower levels of Paraoxonase-1 (PON1) and brain-derived neurotrophic factor (BDNF), both of which have known antioxidant and anti-inflammatory properties, in hippocampal and striatal tissues. When compared with the reserpine-treated group, one-way ANOVA revealed that L-Dopa, BV, DA-nanoparticles significantly reduced the levels of proinflammatory cytokines (TNF-α and IL-1β) and increased the level of endogenous antioxidant (PON1) and stabilized neurotrophic factor (BDNF) in both striatum and hippocampus. However, the inflammatory mediators (TNF-α, IL-1β, PON1) and neurotropic factor (BDNF) were significantly different from the control group (Fig. 3). Our results suggest that L-Dopa, BV, DA-nanoparticles may exhibit neuroprotective properties via inducing anti-inflammatory and antioxidant actions in brain tissues.

### Effects of L-Dopa, BV, DA-nanoparticles on reserpine-induced DNA damage in rat model of PD using DNA Comet assay

**Figure 4:**
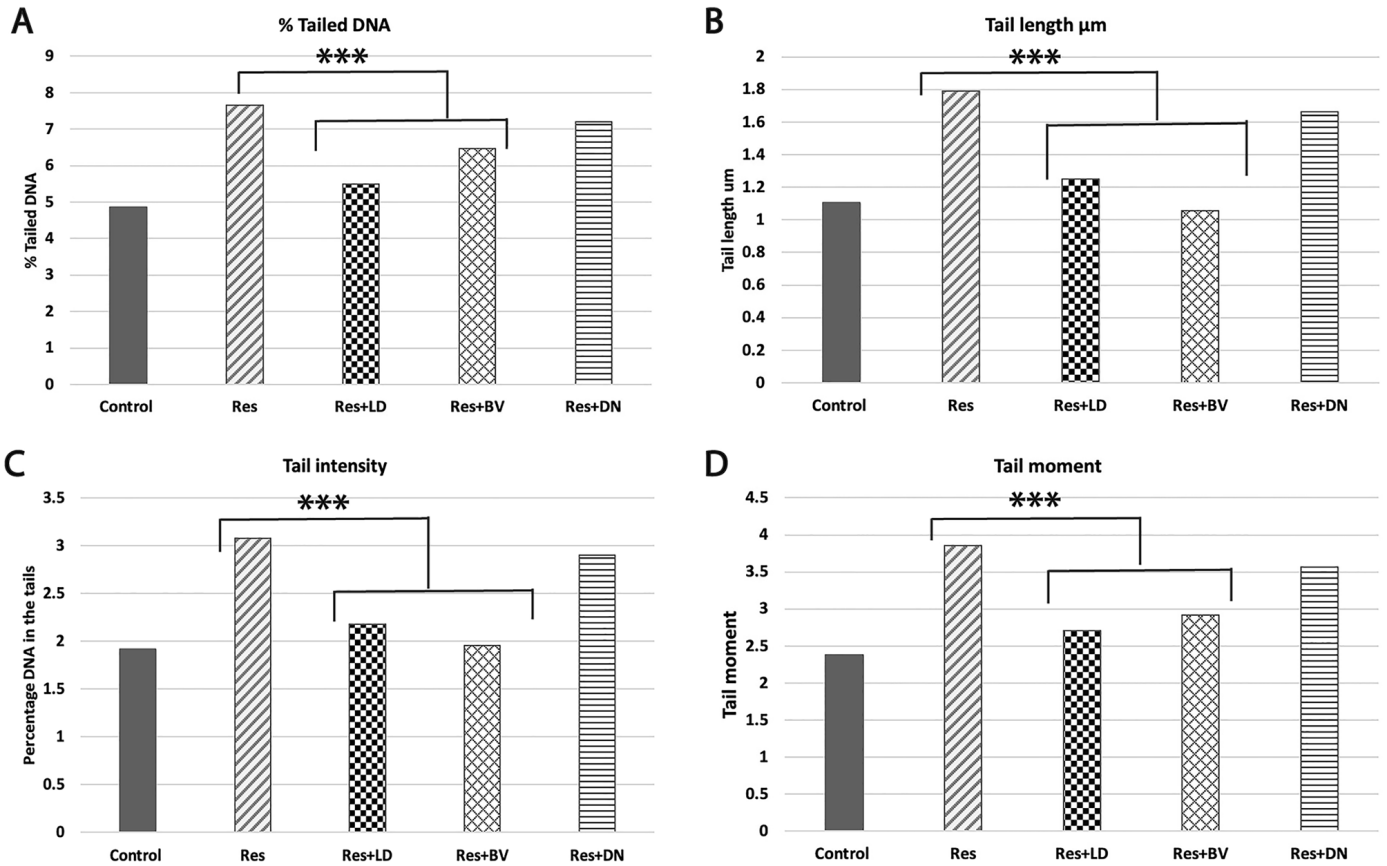
Effects of L-Dopa, BV, DA-nanoparticles against reserpine-induced DNA damage using DNA Comet assay. (**A**) % of tailed DNA, (**B**) tail length in um, (**C**) tail intensity that indicates the percentage of DNA in the tail, (**D**) tail moment.

Using the comet assay, we investigated the effect of L-Dopa, BV, DA-nanoparticles on the induction of DNA strand-breaks in the hippocampus and striatum of a reserpine-induced PD rat model. When compared with control group, DNA comet assay of rats treated with reserpine demonstrated significant increase in DNA damage in the hippocampus and striatum as detected by the % of tailed DNA, tail length, the percentage of DNA in the tail, and the tail moment (Fig. 4A–D).

Next, we assessed the effects of L-Dopa (22.5 mg/kg, P.O.), BV (10 μl/kg b.w i.p.), and DA-nanoparticles (22.5 mg/kg P.O.) administration for 30 days on reserpine-induced DNA damage in the brain. Our results demonstrated significant decrease (*p* < 0.05) in the % of tailed DNA, tail length, the percentage of DNA in the tail, and the tail moment after treatment with both L-Dopa and BV when compared with reserpine-treated group. When comparing the DA-nanoparticles-treated group with reserpine-treated group, For DA-nanoparticles-treated group, no significant difference was detected in all parameters. Our results indicate the protective effect of both L-Dopa and BV against reserpine-induced DNA damage in both hippocampus and striatum in rat model of PD (Fig. 4A–D).

## Discussion

PD is a disabling central nervous system disorder, involving primarily a selective loss of dopaminergic neurons in the substantia nigra; a key neuropathological hallmark which causes motor dyscoordination^35^. Currently, prevention or early treatment of PD can be the most effective therapeutic strategy^36^. Antiparkinsonian medications like levodopa and carbidopa replenish brain dopamine and decrease PD activity. However, these drugs are only symptom-targeting, their effectiveness decreases as the disease progresses and more motor complications develop. These drawbacks promoted researchers to look for new strategies to treat PD, including complementary and alternative treatments. Therefore, this study was conducted to evaluate the effect of BV and DA-nanoparticles on reserpine-induced animal model of PD and compare their effects with L-Dopa, as a conventional therapy of PD. Our findings revealed an evidence for the ameliorative impact of BV and DA-nanoparticles against reserpine-evoked PD by restoring monoamines and BDNF levels and eliciting coordinated anti-inflammatory and antioxidant actions. Of note, the ameliorative effects of BV were comparable to that of L-Dopa when administered to reserpine-induced PD rat model.

In this study, we employed a reserpine-induced animal model of PD that reflect aspects of PD neuropathology and mimics the human PD^23,37^. Reserpine, a potent VMAT2 inhibitor, induces muscular rigidity, tremors, and akinesia by depletion of central catecholamine storage^23^. In the current study, reserpine provoked a neurotransmitters imbalance in the striatum and hippocampus, as demonstarted by monoamines depletion, elevated the levels of excitatory amino acids (Glu and His), diminished inhibitory amino acids (GABA and Arg), and decreased levels of BDNF. These observed imbalances in neurotransmitters and the brain derived neurotropic factor have previously been demonstarted to cause akinesia and paralysis syndrome in rats, which is analogous to human PD^38,39^.

While the aetiology of PD is unclear, oxidative stress and neuroinflammation have been identified as two key entry points in the pathogenesis of the disease via inducing neurodegeneration^40^. Our results demonstrated increased inflammatory response marked with elevated levels of proinflammatory cytokines IL-1β and TNF-α in both the hippocampus and the striatum of rats administered reserpine-alone. These results are in agreement with other studies reported increased neuroinflammatory markers and oxidative stress in different experimental models of PD^38,41–43^. Increased inflammatory responses, combined with T cell infiltration into the brain and activation of microglial cell, have been shown to produce an excess of pro-inflammatory cytokines, causing mitochondrial dysfunction and the production of reactive oxygen species (ROS), which aggravate nerve damage in PD^42,44,45^. In this context, reserpine caused elevation of IL-1β, which binds to IL-1 receptors and produces ROS, which then activates of the NF-κB signalling pathway, which is consistent with our results. Moreover, inflammatory processes produce an excess of ROS, which causes oxidative damage to dopaminergic neurons^46^. On the other hand, we found that reserpine lowered the activity of the antioxidant neuron marker PON1, which was consistent with prior results^47^. PON1 enzyme is well known for its ability to protect the membranes from lipid peroxidation caused by high density lipoprotein^48^. This unequivocal oxidative state of the brain indicates reduction of the cellular antioxidant capacity inevitably leading to cell disruption and DNA damage, which mimics the situation in the PD brain. This was showed in our study by increased DNA fragmentation in reserpine-alone treated rats.

Similar to standard treatment with L-dopa, our findings demonstrated that treatment with either BV or DA-nanoparticles increased the levels of monoamines neurotransmitters like dopamine, norepinephrine, serotonin, and ACh. The observed elevation of NE in our results could be a side consequence of elevated dopamine, the main precursor for norepinephrine synthesis. This is consistent with previous studies reported the restoration of dopamine levels in the hippocampus and striatum in various rat models of PD^34,38^. In a rotenone model of PD in rats, the pharmacological effects of BV and DA-nanoparticles were previously demonstrated to improve motor function^38^. Furthermore, BV acupuncture was found in another study to reverse the depletion of both dopamine and norepinephrine in mice after rotenone intoxication^49^. In addition, in 6-OHDA-induced parkinsonian rats, systemic intravenous administration of DA-nanoparticles markedly boosted dopamine and its metabolites, reduced dopamine-D2 receptor super sensitivity in the striatum, and recovered neurobehavioral deficits^34^. The restoration of monoamines by DA-nanoparticles could be explained the ability of these nanoparticles to cross the blood brain barrier and capillary endothelium in the striatum and substantia nigra and were internalized in dopaminergic neurons, as previously reported in a 6-hydroxydopamine-induced rat model of PD^34^.

Another interesting finding is that both L-Dopa and BV were able to ameliorate neuroexcitation in both brain areas as evidenced by increased GABA and Arg levels and reduced Glu levels in treated groups. The restoration of GABA and Arg, as well as the reduction in Glu, were not significant upon administration of DA-nanoparticles. This is in the line of the findings of El-Ansary et al. who reported that bee pollen ameliorated glutamate excitotoxicity, enhanced the GABA levels, and improved the glutamine/glutamate/GABA circuit in propionic acid-treated rats^50^. Furthermore, the elevated levels of arginine in the striatum and hippocampus following BV administration could possibly enhances nitric oxide synthesis, which is a key signal in neuronal transmission^51^. These results delineate the therapeutic potential of BV as supplemental or alternative treatment for PD. Moreover, our results highlighted that BV and L-Dopa outperformed DA-nanoparticles in protecting against reserpine-induced neurotoxicity.

Similar to conventional treatment with L-Dopa, our results indicated that administration of BV and DA-nanoparticles alleviated neuroinflammation and oxidative stress in examined brain tissues, as detected by decreased IL-1β and TNF-α along with elevated PON1 levels. This is consistent with previous observations by others who reported that both BV and DA-nanoparticles exerted anti-inflammatory and antioxidant actions in different PD rat models^34,38,43^. Our findings are also in line with two other studies which reported that BV acupuncture had antioxidant effects by lowering PON1 enzymatic activity and protecting dopaminergic neurons from oxidative damage in distinct animal model of PD^49,52^. Both anti-inflammatory and antioxidant effects of BV can explain our findings of reduced DNA fragmentation pattern in the comet assay of treated rats. The anti-inflammatory actions of BV could be exerted by melittin and PLA2, two of its major components^17^. Melittin and PLA2, both produced from BV, have been shown to have therapeutic potential to neurodegenerative and chronic inflammatory diseases^53–55^. Indeed, BV is known to exert anti-inflammatory activity in microglial cells via different pathways as following: (i) targeting IRAK1/TAK1/NF-*κ*B signalling pathways, (ii) reducing the mRNA expression of COX-2, TNF-α, IL-1β, and IL-6, (iii) suppressing lipopolysaccharide (LPS)-induced activation of MyD88 and IRAK1, (iv) attenuating NF-*κ*B translocation through decreasing IKK*α*/*β* phosphorylation and subsequent I*κ*B-*α* degradation^56^. Furthermore, BV and its components were found to alter electrical signals transduction between neurons by modulating the ion pumps and channels activities^17,57,58^. Together, our results imply the potential utility of BV and DA-nanoparticles as alternative or complementary therapies of PD. In reserpine-induced PD rat model, however, the revealed ameliorative effects of BV were significantly superior to that of DA-nanoparticles.

Overexpression of neurotrophic factors is a potent method for preventing the dopaminergic neurons loss in PD brains^36^. Brain derived neurotrophic factor (BDNF) plays a crucial role in synaptic plasticity, neuronal survival and proliferation through binding to its TrkB receptor which signals through MAPK, PI3K/Akt and PLC-γ pathways to enhance CREB expression, resulting in neuroregeneration and neuroprotection^59^. Moreover, overexpression of BDNF in animal models of PD, improves the survival of dopaminergic neurons by enhancing dopaminergic cell lifespan via anti-apoptotic activity^60^. Consistent with these findings, the BDNF level was restored in the hippocampus and the striatum of reserpine treated rats after administration of BV and DA nanoparticles in the current study, with more prominent action of BV which normalized BDNF levels in both brain regions. This could be a novel mechanism for BV and DA-nanoparticles to promote neuroprotection and neuroregeneration in PD brains by increasing BDNF levels in the hippocampus and striatum.

In conclusion, our findings revealed that both BV and DA-nanoparticles exhibit neuroregenerative and neuroprotective effects in rats with reserpine-induced PD. These actions are mediated by several concerted mechanisms, including anti-inflammatory, antioxidant, and neurotropic effects. Indeed, in reserpine-induced PD rat model, our results demonstrated that the ameliorative effects of BV were significantly superior to those of DA-nanoparticles. These findings point to the potential of BV and DA nanoform as effective adjuvant treatments for PD.
