# Author Correction: Effects of bee venom and dopamine-loaded nanoparticles on reserpine-induced Parkinson’s disease rat model

**DOI:** 10.1038/s41598-021-01408-x

**Published:** 2021-11-01

**Authors:** Omar A. Ahmed‑Farid, Mohamed Taha, Rofanda M. Bakeer, Omyma K. Radwan, Hassan A. M. Hendawy, Ayman S. Soliman, Einas Yousef

**Affiliations:** 1 Physiology Department, National Organization for Drug Control and Research (NODCAR), Giza, Egypt; 2 Biochemistry Department, Faculty of Pharmacy, Cairo University, Giza, Egypt; 3 Pathology Department, Faculty of Medicine, Helwan University, Helwan, Egypt; 4 Analytical and Inorganic Chemistry Department, NODCAR, Giza, Egypt; 5 Medical Physiology Department, Faculty of Medicine, Beni-Suef University, Beni‑Suef, Egypt; 6 Basic Medical Sciences Department, College of Medicine, Dar Al Uloom University, Riyadh, Kingdom of Saudi Arabia; 7 Histology and Cell Biology Department, Faculty of Medicine, Menoufia University, Shibin El Kom, Egypt

Correction to: *Scientific Reports* https://doi.org/10.1038/s41598-021-00764-y, published online 27 October 2021

The original version of this Article contained an error in the spelling of the author Ayman S. Soliman which was incorrectly given as Ayman Samaan. The original Article has been corrected.

